# Overlap Syndrome Consisting of Polymyositis/Dermatomyositis and ANCA-Associated Vasculitis According to the 2022 ACR/EULAR Criteria for Vasculitis: A Korean Single-Centre Study

**DOI:** 10.3390/jcm12216748

**Published:** 2023-10-25

**Authors:** Jang Woo Ha, Yong-Beom Park, Sang-Won Lee

**Affiliations:** 1 Division of Rheumatology, Department of Internal Medicine, Yonsei University College of Medicine, Seoul 03722, Republic of Korea; hjwnmk@yuhs.ac (J.W.H.); yongbpark@yuhs.ac (Y.-B.P.); 2 Institute for Immunology and Immunological Diseases, Yonsei University College of Medicine, Seoul 03722, Republic of Korea

**Keywords:** antineutrophil cytoplasmic antibody, vasculitis, criteria, polymyositis, dermatomyositis

## Abstract

The present study applied the 2022 American College of Rheumatology and European Alliance of Associations for Rheumatology classification criteria (the 2022 ACR/EULAR criteria) for antineutrophil cytoplasmic antibody (ANCA)-associated vasculitis (AAV) to ANCA-positive patients with polymyositis (PM)/dermatomyositis (DM). Also, this study investigated how many patients could be diagnosed with overlap syndrome consisting of PM/DM and AAV. Twelve ANCA-positive patients with PM/DM were included and analysed in this study. The 2022 ACR/EULAR classification criteria for microscopic polyangiitis (MPA), granulomatosis with polyangiitis (GPA), and eosinophilic GPA (EGPA) are scoring systems, and when a total score is over five in cases of MPA and GPA and over six in cases of EGPA, AAV can be classified. The median age of 12 ANCA-positive patients (six with PM and six with DM) was 54.0 years and one patient (8.3%) was male. Of the 12 ANCA-positive patients with PM/DM, 11 had myeloperoxidase (MPO)-ANCA (or perinuclear [P]-ANCA) and the remaining one had proteinase 3 (PR3)-ANCA (or cytoplasmic [C]-ANCA). Nine (75.5%) and one (8.3%) ANCA-positive patients with PM/DM were diagnosed with overlap syndrome consisting of PM/DM and MPA and overlap syndrome consisting of PM/DM and GPA, respectively. The main contributors to the classification of MPA and GPA were interstitial lung disease and renal vasculitis, along with ANCA positivity, respectively. In conclusion, this study was the first to demonstrate that 83.3% of them could be diagnosed with overlap syndrome consisting of PM/DM and AAV according to the 2022 ACR/EULAR criteria for AAV.

## 1. Introduction

Antineutrophil cytoplasmic antibody (ANCA)-associated vasculitis (AAV) is a small-vessel vasculitis characterised by necrotising vasculitis with few or no immune deposits in small vessels and medium-sized arteries [1]. To date, AAV has been classified based on the following three methods: (i) the 1990 American College of Rheumatology (ACR) criteria for the classification of Churg-Strauss syndrome [2], (ii) the 2007 European Medicine Agency classification of ANCA-associated vasculitides and polyarteritis nodosa (the 2007 EMA algorithm) [3], and (iii) the 2012 revised International Chapel Hill Consensus Conference Nomenclature of Vasculitides [1]. In March 2022, a group of the ACR and European Alliance of Associations for Rheumatology (EULAR) proposed the new classification criteria (the 2022 ACR/EULAR criteria) for microscopic polyangiitis (MPA) [4], granulomatosis with polyangiitis (GPA) [5], and eosinophilic GPA (EGPA) [6]. The new criteria have three distinct altered conditions: the first is that a new scoring system was introduced; the second is that the entry requirements, including clinical features suggestive of vasculitis and the exclusion of other alternate diagnoses mimicking vasculitis should be met; and the third is that the clinical weight of ANCA positivity should be increased compared to the previous criteria [4,5,6].

The third condition motivated us to determine the clinical significance of ANCA positivity among ANCA-positive patients classified as having polymyositis (PM) and dermatomyositis (DM) based on both the Bohan and Peter criteria for PM/DM and the 2017 EULAR/ACR classification criteria for idiopathic inflammatory myopathy [7,8,9]. In clinical settings, when a patient with PM has myeloperoxidase (MPO)-ANCA and interstitial lung disease (ILD), the patient can be classified as having overlap syndrome consisting of PM and MPA [4]. Also, if a patient with DM has proteinase 3 (PR3)-ANCA and paranasal sinusitis, can the patient be classified as having overlap syndrome consisting of DM and GPA [5]? To answer this question, the present study investigated the number of patients diagnosed with overlap syndrome consisting of PM/DM and AAV by applying the 2022 ACR/EULAR criteria to ANCA-positive patients with PM/DM.

## 2. Materials and Methods

### 2.1. Patients

The present study retrospectively reviewed the medical records of 75 PM/DM patients based on the following inclusion criteria: (i) the diagnosis of PM/DM was made at the Division of Rheumatology, Department of Internal Medicine, Yonsei University College of Medicine, Severance Hospital, between January 2005 and December 2021; (ii) the fulfilment of both the Bohan and Peter criteria for probable and definite PM/DM and the 2017 EULAR/ACR classification criteria for probable and definite IIM (Supplementary Table S1) [7,8,9]; (iii) the positive results of ANCA at the time of PM/DM diagnosis; (iv) no concomitant serious medical conditions affecting the classification of PM/DM and AAV such as malignancies and severe infectious diseases requiring hospitalisation at PM/DM diagnosis; (v) no exposure to glucocorticoids or immunosuppressive drugs before PM/DM diagnosis; and (vi) no drug history affecting ANCA results such as propylthiouracil or hydralazine [10]. Finally, 12 ANCA-positive patients classified as having PM/DM who met the inclusion criteria were included and analysed in the present study. This study was approved by the Institutional Review Board (IRB) of Severance Hospital (Seoul, Republic of Korea; IRB No. 4-2021-1057) and conducted in accordance with the Declaration of Helsinki. Given the retrospective study design and the use of anonymized patient data, the requirement for written informed consent was waived by the IRB.

### 2.2. Clinical Data

Age and sex were collected as demographic data. The numbers of patients fulfilling each item of the Bohan and Peter criteria and the 2017 EULAR/ACR classification criteria for IIM were counted. According to the 2022 ACR/EULAR criteria for AAV [4,5,6], perinuclear (P)-ANCA and cytoplasmic (C)-ANCA positivity were approved in addition to MPO-ANCA and PR3-ANCA positivity. In our hospital, the tests for ANCAs are routinely performed in patients suspected of autoimmune connective tissue diseases at the first time of visit. An indirect immunofluorescence assay for P-ANCA and C-ANCA is first performed, and then an immunoassay for MPO-ANCA and PR3-ANCA is performed when P-ANCA or C-ANCA is detected. ILD was confirmed by high-resolution computed tomography (HRCT). The variables of the remaining laboratory results, including the PM/DM-specific results are summarised in Table 1. 

### 2.3. The 2022 ACR/EULAR Criteria for AAV

The 2022 ACR/EULAR classification criteria for MPA, GPA, and EGPA are scoring systems comprising two major criteria: clinical criteria and laboratory, imaging, and biopsy criteria. Different weighted points are assigned to each item, and when the total score is over 5 in cases of MPA and GPA and over 6 in cases of EGPA, AAV can be classified [4,5,6].

### 2.4. Statistical Analyses

Continuous variables were expressed as medians with interquartile ranges, whereas categorical variables were expressed as numbers (percentages).

## 3. Results

### 3.1. Characteristics of Patients at PM/DM Diagnosis

Of the 12 ANCA-positive patients with PM/DM, six had PM and the remaining six had DM. The median age was 54.0 years and one patient (8.3%) was male. Histological findings on muscle biopsy were assessed in 11 of the 12 patients with PM/DM. A total score of the 2017 EULAR/ACR criteria for IIM was 7.9, which was over the cut-off for probable IIM (≥5.5 without muscle biopsy and ≥6.7 with muscle biopsy). The median AST, ALT, CPK, LDH and aldolase were 73.0 IU/L, 38.0 IU/L, 380.0 IU/L, 434.0 IU/L and 12.3 (sigma u/mL), respectively. Of the 12 ANCA-positive patients with PM/DM, 11 had MPO-ANCA (or P-ANCA) and the remaining patient had PR3-ANCA (or C-ANCA). The remaining laboratory results are shown in Table 1.

### 3.2. Application of the 2022 ACR/EULAR Criteria for MPA to ANCA-Positive Patients Classified as Having PM/DM at the Time of Diagnosis

In terms of clinical criteria, none of the patients exhibited nasal involvement. In terms of laboratory, imaging, and biopsy criteria, the most frequent item was MPO-ANCA (or P-ANCA) positivity (91.7%), followed by ILD on chest imaging (75.0%). Conversely, a score of −1 and −4 were given to one and two patients owing to PR3-ANCA (or C-ANCA) positivity and serum eosinophil count ≥ 1000/μL, respectively. Nine of the 12 ANCA-positive patients with PM/DM (75.0%) achieved a total score ≥ 5 and could be diagnosed with overlap syndrome consisting of PM/DM and MPA (Table 2). 

### 3.3. Application of the 2022 ACR/EULAR Criteria for GPA to ANCA-Positive Patients Classified as Having PM/DM at the Time of Diagnosis

Regarding clinical criteria, none presented with nasal, cartilaginous, or hearing problems. Regarding laboratory, imaging, and biopsy criteria, the most common item was nasal/paranasal sinusitis, or mastoiditis on imaging (33.3%). Eleven and two patients were scored −1 and −4 due to MPO-ANCA (or P-ANCA) positivity and serum eosinophil count ≥1000/μL, respectively. Only one of the 12 ANCA-positive patients with PM/DM (8.3%) achieved a total score ≥ 5 and could be diagnosed with overlap syndrome consisting of PM/DM and GPA (Table 3). 

### 3.4. Application of the 2022 ACR/EULAR Criteria for EGPA to ANCA-Positive Patients Classified as Having PM/DM at the Time of Diagnosis

In terms of clinical criteria, only one patient exhibited clinical features of obstructive airway disease, but none had nasal polyps or neuropathy. In terms of laboratory, imaging, and biopsy criteria, two patients showed an increased serum eosinophil count. Five patients had −1 due to haematuria and one patient had −3 due to PR-3 ANCA (or C-ANCA) positivity. None of the 12 ANCA-positive patients with PM/DM were diagnosed with overlap syndrome consisting of PM/DM and EGPA (Table 4).

### 3.5. Itemized Analysis of Patients with the Overlap Syndrome of PM/DM and MPA/GPA

Regarding nine ANCA-positive patients diagnosed with overlap syndrome consisting of PM/DM and MPA, all nine patients had MPO-ANCA (or P-ANCA) positivity. Eight patients presented with ILD on chest imaging, and one of them had histopathological features of pauci-immune glomerulonephritis, on biopsy in addition to MPO-ANCA (or P-ANCA) positivity and pulmonary features. One patient without ILD had haematuria and proteinuria 2+ on a urine stick, which was defined as renal vasculitis by the 2007 EMA algorithm and was considered evidence of small-vessel vasculitis by the 2022 ACR/EULAR criteria. Therefore, this patient achieved a total score of 6 because of MPO-ANCA (or P-ANC A) positivity and was diagnosed with overlap syndrome consisting of PM/DM and MPA. Whereas, regarding one ANCA-positive patient diagnosed with overlap syndrome consisting of PM and GPA, this patient had PR3-ANCA (or C-ANCA) and ILD but not pulmonary nodules or cavitation suggestive of GPA. Nevertheless, since ILD was considered evidence of small-vessel vasculitis, this patient achieved a total score of 5 because of PR3-ANCA (or C-ANCA) positivity and was diagnosed with overlap syndrome consisting of PM and GPA (Table 5)

## 4. Discussion

The present study is the first to demonstrate that 83.3% of the 12 patients with PM/DM in whom ANCA was detected at the time of PM/DM diagnosis could be diagnosed with overlap syndrome consisting of PM/DM and AAV according to the 2022 ACR/EULAR criteria for AAV. 

First, in terms of nine patients diagnosed with overlap syndrome consisting of PM/DM and MPA, the most critical contributor to the classification of MPA was ILD because ILD was found in eight of the nine patients. Nevertheless, at the time of PM/DM diagnosis, it is unlikely that MPA could have been naturally considered in these eight patients with MPO-ANCA (or P-ANCA) and ILD. First, before the proposal of the 2022 ACR/EULAR criteria for MPA, ILD was not mentioned in the 2007 EMA algorithm or the 2012 CHCC definitions [1,3]. Second, ILD may have been considered a systemic complication of PM/DM [11]. However, given the muscle involvement of systemic vasculitis [12], we suggest that even when patients can be classified as having PM/DM, the 2022 ACR/EULAR criteria for MPA should be applied simultaneously to patients with MPO-ANCA (or P-ANCA) and ILD. 

In terms of ILD, even though it may not be possible to discriminate whether ILD is a clinical feature of MPA or of PM/DM, why should we focus on the diagnosis of overlap syndrome consisting of PM/DM and MPA in nine patients having MPO-ANCA (or P-ANCA) and ILD? We would like to suggest the following three reasons for the differential diagnoses. First, the consensus-based and recommended remission induction and maintenance therapeutic regimens may be different between patients classified as having only PM/DM and those diagnosed with overlap syndrome consisting of PM/DM and MPA [13,14]. Second, the magnitude of the impact of ILD on prognosis may be different between the two groups [15,16,17]. Third, the types of organs and symptoms to be monitored intensively during the follow-up period may be different between the two groups [18,19,20]. Therefore, we also suggest that even when patients could be classified as having PM/DM, the 2022 ACR/EULAR criteria for MPA should be applied to patients who have MPO-ANCA (or P-ANCA) and ILD as always as possible.

In terms of renal manifestation suggesting small-vessel vasculitis, two of the nine patients diagnosed with overlap syndrome consisting of PM/DM and MPA presented haematuria and proteinuria 2+ on a urine stick, which was consistent with the definition of renal vasculitis described in the 2007 EMA algorithm [3]. One of the two patients with renal vasculitis underwent renal biopsy, which revealed pauci-immune glomerulonephritis [1]; however, since the amount of proteinuria was normalised, the therapy for renal vasculitis was not added and MPA diagnosis might have been ignored. On the other hand, another patient with renal vasculitis exhibited significant proteinuria, a but renal biopsy was not performed because the amount of proteinuria per 24 h was not more than 1 g. Unlike the definition of renal vasculitis in the 2007 EMA algorithm [3], the 2022 ACR/EULAR criteria for AAV recognise only histological findings as significant renal involvement of AAV [4,5]. Therefore, we suggest that renal biopsy should be considered in PM/DM patients in whom MPO-ANCA (or P-ANCA) is detected at the time of PM/DM diagnosis. Furthermore, we also suggest paying persistent attention to those exhibiting pauci-immune glomerulonephritis on renal biopsy.

Second, in terms of one patient diagnosed with overlap syndrome consisting of PM and GPA, the patient had ILD along with PR3-ANCA (or C-ANCA). In this case, ILD did not contribute to the classification of GPA but played a role as evidence of small-vessel vasculitis, which enabled us to apply the 2022 ACR/EULAR criteria for MPA, GPA, and EGPA [4,5,6]. The patient was diagnosed with overlap syndrome consisting of PM and GPA due to only PR3-ANCA (or C-ANCA) positivity. If ILD was nothing but a systemic complication of PM [17], and ANCA was transiently positive, the diagnosis of GPA could not be made. If there had been histological features of GPA on a lung biopsy and PR3-ANCA (or C-ANCA) had been serially measured, the confirmative diagnosis could have been made. Therefore, we suggest that both a lung biopsy and the test of PR3-ANCA (or C-ANCA) should be serially performed in PM/DM patients who have PR3-ANCA (or C-ANCA) and only ILD among systemic manifestations.

The present study has an advantage in that this was the first to apply the 2022 ACR/EULAR criteria for AAV to ANCA-positive patients classified as having PM/DM at the time of diagnosis and demonstrate that 83.3% of them could be diagnosed with overlap syndrome consisting of PM/DM and AAV. However, the present study has several limitations. The number of ANCA-positive patients classified as having PM/DM was insufficient to generalise the results of this single-centre study. In addition, since the present study was designed as a retrospective study, all data at the time of diagnosis had to depend on the medical records and thus the accuracy of the diagnosis might be reduced. Additionally, we could not request a test for several IIM-specific antibodies, which was performed in the outside institute due to a retrospective study design. Given the ethnic and geographical differences, another limitation was that only Korean patients were investigated in the present study. Nevertheless, the present study has the clinical implications as the first pilot study, and it is believed that future prospective studies with a larger number of ANCA-positive PM/DM patients and those with a serial ANCA measurement will validate the results of this study and enhance its reliability.

## 5. Conclusions

The present study applied the 2022 ACR/EULAR criteria for AAV to 12 ANCA-positive patients classified as having PM/DM and demonstrated that 83.3% of them could be diagnosed with overlap syndrome consisting of PM/DM and AAV. Therefore, we suggest that the 2022 ACR/EULAR criteria for AAV should be applied to PM/DM patients who had MPO-ANCA (or P-ANCA) or PR3-ANCA (or C-ANCA) along with any evidence of small- and medium-vessel vasculitis. Also, we suggest that aggressive biopsy and serial ANCA tests should be performed in PM/DM patients who had PR3-ANCA (or C-ANCA) and evidence of small-vessel vasculitis in the lungs and kidneys.

## Figures and Tables

**Table 1:** Characteristics of ANCA-positive patients with PM/DM at the time of diagnosis (*n* = 12).

Variables	Values
*At the time of diagnosis*	
Subtype	
PM	6 (50.0)
DM	6 (50.0)
Demographic data	
Age (years)	54.0 (9.0)
Male sex (*n*, (%))	1 (8.3)
Items of the Bohan and Peter criteria	
Symmetrical proximal muscle weakness	10 (83.3)
Muscle biopsy consistent with PM/DM	9/11 (81.8)
Elevated muscle enzyme	11 (91.7)
Abnormal EMG	8 (66.7)
Dermatologic features suggestive of DM	7 (58.3)
Items of the 2017 EULAR/ACR criteria for IIM	
Age of onset of first symptom18–40 years	1 (8.3)
Age of onset of first symptom ≥40 years	11 (91.7)
Objective symmetric weakness, usually progressive, of the proximal upper extremities	6 (50.0)
Objective symmetric weakness, usually progressive, of the proximal lower extremities	11 (91.7)
Neck flexors are relatively weaker than neck extensors	2 (16.7)
In the legs, proximal muscles are relatively weaker than distal muscles	9 (75.0)
Heliotrope rash	1 (8.3)
Gottron’s papules	3 (25.0)
Gottron’s sign	5 (41.7)
Dysphagia or oesophageal dysmotility	0 (0.0)
Anti-Jo-1 antibody positivity	1 (8.3)
Elevated serum levels of CPK or LDH or AST or ALT	11 (91.7)
Endomysial infiltration of mononuclear cells surrounding, but not invading, myofibers	3/11 (27.3)
Perimysial and/or perivascular infiltration of mononuclear cells	5/11 (45.5)
Perifascicular atrophy	1/11 (9.1)
Rimmed vacuoles	0/11 (0.0)
Total score	7.9 (4.7)
Laboratory findings (reference ranges)	
White blood cell count (/mm^3^) (4000.0–10,800.0)	5390.0 (1910)
Haemoglobin (g/dL) (13.0–17.4)	11.9 (0.9)
Platelet count (10^3^/mm^3^) (150.0–400.0)	229.0 (65.0)
Blood urea nitrogen (mg/dL) (8.5–22.0)	11.7 (6.9)
Serum creatinine (mg/dL) (0.67–1.2)	0.5 (0.3)
AST (IU/L) (13.0–34.0)	73.0 (176.0)
ALT (IU/L) (5.0–46.0)	38.0 (140.0)
Total protein (g/dL) (6.0–8.0)	6.6 (0.8)
Serum albumin (g/dL) (3.3–5.3)	3.6 (0.6)
CPK (IU/L) (44.0–245.0)	380.0 (3226.0)
LDH (IU/L) (119.0–247.0)	434.0 (587.0)
Aldolase (sigma u/mL) (0–7.6)	12.3 (55.9)
ANCA positivity (*n*, (%))	
ANCA positivity	12 (100.0)
MPO-ANCA (or P-ANCA) positivity	11 (91.7)
PR3-ANCA (or C-ANCA) positivity	1 (8.3)

Values are expressed as a median (25–75 percentiles) or a number (percentage). PM/DM: polymyositis/dermatomyositis; EMG: Electromyography; EULAR: the European Alliance of Associations for Rheumatology; ACR: the American College of Rheumatology; IIM: idiopathic inflammatory myopathy; CPK: creatinine phosphokinase; LDH: lactate dehydrogenase; AST: aspartate aminotransferase; ALT: alanine aminotransferase; ANCA: antineutrophil cytoplasmic antibody; MPO: myeloperoxidase; P: perinuclear; PR3: proteinase 3; C: cytoplasmic.

**Table 2:** Application of the 2022 ACR/EULAR criteria for MPA to ANCA-positive patients classified as having PM/DM at the time of diagnosis.

Variables		Values
*At the time of diagnosis*	*Score*	
Items for the 2022 ACR/EULAR criteria for MPA and assigned scores to each item (*n* (%))		
Clinical criteria		
Nasal involvement (discharge, ulcers, crusting, congestion, septal defect/perforation)	−3	0 (0.0)
Laboratory, imaging, and biopsy criteria		
MPO-ANCA (or P-ANCA) positivity	+6	11 (91.7)
Fibrosis or interstitial lung disease on chest imaging	+3	9 (75.0)
Pauci-immune glomerulonephritis on biopsy	+3	1 (8.3)
PR3-ANCA (or C-ANCA) positivity	−1	1 (8.3)
Serum eosinophil count ≥ 1000/μL	−4	2 (16.7)
Total score for 6 items above		6.0 (6.0)
Patients with total score ≥ 5 (*n* (%))		9 (75.0)

Values are expressed as a number (percentage). ACR: the American College of Rheumatology; EULAR: the European Alliance of Associations for Rheumatology; MPA: microscopic polyangiitis; PM: polymyositis; DM: dermatomyositis; MPO: myeloperoxidase; P: perinuclear; PR3: proteinase 3; C: cytoplasmic.

**Table 3:** Application of the 2022 ACR/EULAR criteria for GPA to ANCA-positive patients classified as having PM/DM at the time of diagnosis.

Variables		Values
*At the time of diagnosis*	*Score*	
Items for the 2022 ACR/EULAR criteria for GPA and assigned scores to each item (*n* (%))		
Clinical criteria		
Nasal involvement (discharge, ulcers, crusting, congestion, septal defect/perforation)	+3	0 (0.0)
Cartilaginous involvement	+2	0 (0.0)
Conductive or sensorineural hearing loss	+1	0 (0.0)
Laboratory, imaging, and biopsy criteria		
PR3-ANCA (or C-ANCA) positivity	+5	1 (8.3)
Pulmonary nodules, mass, or cavitation	+2	1 (8.3)
Granuloma, granulomatous inflammation, or giant cells on biopsy	+2	0 (0.0)
Nasal/paranasal sinusitis or mastoiditis on imaging	+1	4 (33.3)
Pauci-immune glomerulonephritis on biopsy	+1	1 (8.3)
MPO-ANCA (or P-ANCA) positivity	−1	11 (91.7)
Serum eosinophil count ≥ 1000/μL	−4	2 (16.7)
Total score for 10 items above		−1.0 (1.0)
Patients with total score ≥ 5 (*n* (%))		1 (8.3)

Values are expressed as a number (percentage). ACR: the American College of Rheumatology; EULAR: the European Alliance of Associations for Rheumatology; GPA: granulomatosis with polyangiitis; PM: polymyositis; DM: dermatomyositis; PR3: proteinase 3; C: cytoplasmic; MPO: myeloperoxidase; P: perinuclear.

**Table 4:** Application of the 2022 ACR/EULAR criteria for EGPA to ANCA-positive patients classified as having PM/DM at the time of diagnosis.

Variables		Values
*At the time of diagnosis*	*Score*	
Items for the 2022 ACR/EULAR criteria for EGPA and assigned scores to each item (*n* (%))		
Clinical criteria		
Obstructive airway disease	+3	1 (8.3)
Nasal polyps	+3	0 (0.0)
Mononeuritis multiplex	+1	0 (0.0)
Laboratory, imaging, and biopsy criteria		
Serum eosinophil count ≥ 1000/μL	+5	2 (16.7)
Extravascular eosinophilic-predominant inflammation on biopsy	+2	0 (0.0)
PR3-ANCA (or C-ANCA) positivity	−3	1 (8.3)
haematuria	−1	5 (41.7)
Total score for 7 items above		0.0 (1.0)
Patients with total score ≥ 6 (*n* (%))		0 (0.0)

Values are expressed as a number (percentage). ACR: the American College of Rheumatology; EULAR: the European Alliance of Associations for Rheumatology; EGPA: eosinophilic granulomatosis with polyangiitis; PM: polymyositis; DM: dermatomyositis; PR3: proteinase 3; C: cytoplasmic.

**Table 5:** Itemized analysis of PM/DM patients who met the 2022 ACR/EULAR criteria for MPA or GPA (*n* = 10).

**Patient’s Number**	**Scores Based on the 2022 ACR/EULAR Criteria for MPA**	**Subtype**	**1** **(−3)**	**2** **(+6)**	**3** **(+3)**	**4** **(+3)**	**5** **(−1)**	**6** **(−4)**					
1	12	DM	0	1	1	1	0	0					
2	9	DM	0	1	1	0	0	0					
3	9	DM	0	1	1	0	0	0					
4	9	DM	0	1	1	0	0	0					
5	9	PM	0	1	1	0	0	0					
6	9	PM	0	1	1	0	0	0					
7	9	PM	0	1	1	0	0	0					
8	9	PM	0	1	1	0	0	0					
9	6	PM	0	1	0	0	0	0					
	1 = Nasal involvement (discharge, ulcers, crusting, congestion, septal defect/perforation); 2 = MPO-ANCA (or P-ANCA) positivity; 3 = Fibrosis or interstitial lung disease on chest imaging; 4 = Pauci-immune glomerulonephritis on biopsy; 5 = PR3-ANCA (or C-ANCA) positivity; 6 = Serum eosinophil count ≥ 1000/μL												
Patients number	Scores based on the 2022 ACR/EULAR criteria for GPA		1 (+3)	2 (+2)	3 (+1)	4 (+5)		5 (+2)	6 (+2)	7 (+1)	8 (+1)	9 (−1)	10 (−4)
10	5	PM	0	0	0	1		0	0	0	0	0	0
	1 = Nasal involvement (discharge, ulcers, crusting, congestion, septal defect/perforation); 2 = Cartilaginous involvement; 3 = Conductive or sensorineural hearing loss; 4 = PR3-ANCA (or C-ANCA) positivity; 5 = Pulmonary nodules, mass or cavitation; 6 = Granuloma, granulomatous inflammation, or giant cells on biopsy; 7 = Nasal/paranasal sinusitis or mastoiditis on imaging; 8 = Pauci-immune glomerulonephritis on biopsy; 9 = MPO-ANCA (or P-ANCA) positivity; 10 = Serum eosinophil count ≥ 1000/μL.												

PM: polymyositis; DM: dermatomyositis; ACR: the American College of Rheumatology; EULAR: the European Alliance of Associations for Rheumatology; MPA: microscopic polyangiitis; GPA: granulomatosis with polyangiitis; MPO: myeloperoxidase; P: perinuclear; PR3: proteinase 3; ANCA: antineutrophil cytoplasmic antibody; C: cytoplasmic.

## Data Availability

The data used to support the findings of this study are included within the article.

## Supplementary Materials

The following supporting information can be downloaded at: https://www.mdpi.com/article/10.3390/jcm12216748/s1, Table S1: The items that satisfy the 2017 EULAR classification criteria for idiopathic inflammatory myositis for each ANCA positive patients with PM/DM (*n* = 12).
