# Supplementary material for: Overlap Syndrome Consisting of Polymyositis/Dermatomyositis and ANCA-Associated Vasculitis According to the 2022 ACR/EULAR Criteria for Vasculitis: A Korean Single-Centre Study
Source: J Clin Med. 2023 Oct 25;12(21):6748. doi: 10.3390/jcm12216748 (PMC10647414; doi:10.3390/jcm12216748)
Supplement: Supplementary file 1 [file jcm-12-06748-s001.zip › jcm-2675160-supplementary.pdf]

Supplementary Table S1. The items that satisfy the 2017 EULAR classification criteria for idiopathic inflammatory myositis for each ANCA positive patients with PM/DM (N=12)

Patients	1	2	3	4	5	6	7	8	9	10	11	12	13	14	15	16
1	0	0	0	0	0	0	0	1	0	0	1	0	0	0	0	7.3
2	0	0	1	1	1	0	0	0	0	0	1	0	1	0	0	8.1
3	0	1	1	0	1	0	0	0	0	0	1	0	1	0	0	7.2
4	0	0	1	0	1	0	0	0	0	0	1	1	0	0	0	7.0
5	0	1	1	0	0	0	0	0	0	0	1	0	1	1	0	7.9
6	0	1	1	0	1	0	1	1	0	1	1	0	0	0	0	16.2
7	0	0	1	0	1	0	0	1	0	0	1	0	0	0	0	8.4
8	0	1	1	1	1	0	0	0	0	0	1	0	0	0	0	7.6
9	1	1	1	0	1	0	1	1	0	0	1	1	0	0	0	13.4
10	0	0	1	0	1	0	0	0	0	0	1	1	0	0	0	7.0
11	0	0	1	0	1	0	0	1	0	0	0	0	1	0	0	8.8
12	0	1	1	0	0	1	1	0	0	0	1	0	1	0	0	11.9

Parameter 1: Age of onset of first symptom18-40 years; 2: Objective symmetric weakness, usually progressive, of the proximal upper extremities. 3: Objective symmetric weakness, usually progressive, of the proximal lower extremities; 4: Neck flexors are relatively weaker than neck extensors; 5: In the legs, proximal muscle are relatively weaker than distal muscles; 6: Heliotrope rash; 7: Gottron's papules; 8: Gottron's sign; 9: Dysphagia or esophageal dysmotility; 10: Anti-Jo-1 antibody positivity; 11: Elevated serum levels of CK or LDH or AST or ALT; 12: Endomysial infiltration of mononuclear cells surrounding, but not invading, myofibers; 13: Perimysial and/or perivascular infiltration of mononuclear cells; 14: Perifascicular strophy; 15: Rimmed vacuoles; 16: Total score of 2017 EULAR classification criteria for idiopathic inflammatory myositis
